# A Weak Neutralizing Antibody Response to Hepatitis C Virus Envelope Glycoprotein Enhances Virus Infection

**DOI:** 10.1371/journal.pone.0023699

**Published:** 2011-08-22

**Authors:** Keith Meyer, Arup Banerjee, Sharon E. Frey, Robert B. Belshe, Ranjit Ray

**Affiliations:** 1 Department of Internal Medicine, Saint Louis University, St. Louis, Missouri, United States of America; 2 Molecular Microbiology & Immunology, Saint Louis University, St. Louis, Missouri, United States of America; Ohio State University, United States of America

## Abstract

We have completed a phase 1 safety and immunogenicity trial with hepatitis C virus (HCV) envelope glycoproteins, E1 and E2, with MF59 adjuvant as a candidate vaccine. Neutralizing activity to HCV genotype 1a was detected in approximately 25% of the vaccinee sera. In this study, we evaluated vaccinee sera from poor responders as a potential source of antibody dependent enhancement (ADE) of HCV infection. Sera with poor neutralizing activity enhanced cell culture grown HCV genotype 1a or 2a, and surrogate VSV/HCV pseudotype infection titer, in a dilution dependent manner. Surrogate pseudotypes generated from individual HCV glycoproteins suggested that antibody to the E2 glycoprotein; but not the E1 glycoprotein, was the principle target for enhancing infection. Antibody specific to FcRII expressed on the hepatic cell surface or to the Fc portion of Ig blocked enhancement of HCV infection by vaccinee sera. Together, the results from *in vitro* studies suggested that enhancement of viral infectivity may occur in the absence of a strong antibody response to HCV envelope glycoproteins.

## Introduction

HCV continues to be a major global public health problem despite significant advances in interferon based treatment. New generation of specific antivirals are entering clinical trials; and the use of combination therapy is likely to diminish the development of resistant variants, and provide effective virus control and eradication. The use of cell culture grown HCV and VSV/HCV pseudotype as a surrogate model have proven to be important tools in understanding the role of virus envelope glycoproteins for interactions with cell surface proteins [1], [2], [3], [4], [5]. The entry stage of viral replication constitutes a target for neutralizing antibodies as well as pharmacologic agents. Several lines of evidence suggest important contributions for the two HCV envelope glycoproteins (E1 and E2) in HCV entry using pseudotype models and include: (i) Initial contact of HCV partly depends on sulfated polysaccharides present on mammalian cells and this contact appears to be stronger with the E2 glycoprotein [2], [3], [5], [6], [7], [8], [9]. (ii) CD81 may play a role in virus infectivity through interaction with E2 [2], [3], [10], [11]. (iii) Virus titer decreases with interruption of LDL-R activity and this may be mediated via an E1 specific interaction [2], [3]. These observations suggest that the two different forms of recombinant HCV envelope glycoproteins (chimeric E1-G/E2-G used in VSV pseudotype generation or unmodified E1–E2 used in HIV or MuLV derived pseudotype) display similar functional profiles, and that more than one cellular protein may be responsible for binding and entry of virus particles into hepatocytes. Claudin-1 (CLDN1) has been shown to act at a post-binding stage of HCV [12], although the precise function of CLDN1 in the orchestration of HCV entry is under investigation. Specific entry factors of HCV, like CD81 or SR-B1, may associate with CLDN1 on the basolateral surface of polarized hepatocytes and facilitate HCV cell to cell spread [13], [14]. A recent report suggests that tyrosine kinases mediate HCV entry by regulating CD81-claudin-1 co-receptor associations and HCV glycoprotein dependent membrane fusion [15]. Thus, multiple cellular proteins and cell surface receptors may be involved in an interaction between HCV and host cells for virus entry.

HCV patient derived glycoproteins exhibit marked differences in susceptibility to serum neutralizing antibodies [16]. HCV may exist in the blood as free virus or complexed with antibodies. A large percentage of sera from chronically HCV infected patients or vaccinee sera bind to HCV envelope glycoproteins, but fail to efficiently neutralize infection, and some of these serum antibodies as well as human monoclonal antibodies enhance pseudotype infectious titer [17], [18], [19], [20], [21]. This enhancement may be due to the presence of non-neutralizing antibodies and/or antibodies of low affinity.

Antibody-dependent enhancement of infection has been observed *in vivo* in animal models and among individuals vaccinated against certain viruses, such as flavivirus (yellow fever, dengue), HIV-1, Ebola virus, and Hantavirus [22], [23]. Increased infection occur both through interactions with Fc receptors, and receptors for complement in different human cell lines [24], [25], [26], [27], [28]. The ability of sera to enhance HIV-1 infection in the presence of complement is associated with a progression towards AIDS [23], [24], [25], [29], and an *in vivo* correlate of increased viral burden and antigenemia in a SIV/macaque model [30]. Viruses elicit antibodies that enhance infectivity through the binding of virus-antibody complexes to cellular Fc receptors via the Fc portion of the antibodies*;* leading to an increase in viral uptake, with an accompanying increase in replication and higher viral titer [31], [32]. Understanding the nature of antigen-antibody interactions, along with the role of the complement system in HCV infection may help to clarify the poor performance of anti-HCV specific immunoglobulins in disease progression.

In this study, we evaluated the modulation of cell culture grown HCV and VSV derived pseudotype infectivity by antibodies obtained from volunteers vaccinated against HCV in the presence or absence of complement. The results indicate that enhancement of viral infectivity may occur in the absence of a strong antibody response to HCV envelope glycoproteins.

## Methods

### Vaccinee sera

A randomized, double-blinded, placebo-controlled study (DMID 01–012) was completed after approval by the Saint Louis University Institutional Review Board. All subjects provided informed consent, and the work was conducted with Institutional Approval (IRB # 15719). Immunizations included 4 doses of purified Chinese hamster ovarian cell–derived full-length recombinant E1/E2 glycoproteins of HCV genotype 1a with the MF59 adjuvant (Novartis) in 3 different volunteer groups. The volunteers in each group were immunized 4 times (at 0, 4, 24, and 48 weeks) with 4, 20, or 100 µg of E1/E2 per vaccine dose and MF59 adjuvant. Sixteen volunteers in each group received vaccine using one of the dosage levels and 4 received a placebo control in each group. Serum samples were collected from the volunteers at different time points (0, 6, 26, 50, and 64 weeks) for testing, heat inactivated, and the nature of the antibody responses were characterized [21], [33]. For this study; serum drawn at week 64 (16 weeks after the fourth immunization) was used to evaluate the potential of non-neutralizing sera to affect HCV titer.

### Generation of HCV in cell culture

HCV genotype 1a (clone H77) or HCV genotype 2a (clone JFH1) were grown in immortalized human hepatocytes (IHH) as previously described [4]. Virus was harvested from cells and clarified by low speed centrifugation, followed by filtration through a 0.45-µm cellulose acetate membrane (Nalgene, Rochester, NY). Cell culture grown HCV (HCVcc) were aliquoted and stored at −70°C for single use. RNA (IU/ml) was quantified by real-time polymerase chain reaction (Prism 7500 real-time thermocycler; ABI) with the use of HCV analyte-specific reagents (ASR, Abbott) from the Pathology Clinical Laboratory at Saint Louis University. Virus titer was measured from cell culture supernatant by fluorescent-focus formation (FFU) assay using HCV specific reagents. HCV titer was typically calculated between 10^4^–10^6^ IU/ml and 10^4^–10^5^ FFU/ml. IU/ml values were normally >10 fold as compared to FFU/ml for HCV genotype 1a, and this difference was higher with HCV genotype 2a generated in cell culture.

### Assay for HCVcc infectivity

To quantify antibody dependent enhancement (ADE) of HCV infection by antibody from viral RNA estimation, a predetermined IU of cell culture grown HCV was exposed to serial dilutions of vaccinee sera and incubated for 1 h at 37°C prior to addition to Huh7 cells. Virus containing supernates were incubated with cells for 3 h at 37^o^C. Cells were rinsed twice and incubated at 37°C for 96 h. Cells were rinsed, lysed, and RNA was isolated using the RNAeasy Miniprep kit (Qiagen). cDNA was generated using a Superscript III kit (Invitrogen), followed by real-time PCR to quantify viral RNA titer [34]. A curve of virus dilutions was used as a comparative control, and 18S RNA was used as an internal control. The reciprocal serum dilution is given as X, where X is 1/X.

Further, HCV (clone JFH1) was used to infect Huh7.5 cells using a predetermined (FFU) number of viral particles as determined by FFU. Briefly, virus supernates were diluted as a pool to cover the entire experiment, separated, and treated individually with vaccinee sera for one hour prior to infection of Huh7.5 cells. Infected cells were incubated for 72 hours at 37^o^C. Cells were washed three times with phosphate-buffered saline (PBS) and stained with antibody specific to HCV core (C750, Thermo Fischer, IL) at a dilution of 1/400 or NS5A (kindly provided by Chen Liu, University of Florida) at a dilution of 1/100. Cells were washed three times with PBS, stained with anti-mouse immunoglobulin (Ig) conjugated with Alexa 568 (Molecular Probes, Eugene, OR), and mounted for fluorescence microscopy. Primary antibodies and secondary antibody-fluorochrome conjugates were titrated for use of optimum dilutions where there was no background fluorescence. DAPI was used to enhance focus by nuclear staining prior to imaging. Fluorescence positive infected cells were observed by microscopy, and counted. A human monoclonal antibody to HCV E2 glycoprotein (CBH5) was used as a neutralizing positive control, and HCV negative sera was used as a further control.

### Blocking FcR interactions

A Fab fragment recognizing the Fc region of immunoglobulin (Sigma, St. Louis, MO) was examined for blocking HCV infectivity. The Fc-specific Fab fragment was added at the time of pseudotype/antibody incubation at a concentration of 1ug/ml. Control virus for this experiment included a duplicate treated with the Fc specific Fab fragment, with no change in virus titer. The remainder of the experiment was carried out as described above. Cell culture grown HCV was diluted to a predetermined FFU for estimation of IU, and exposed to vaccinee sera in the presence or absence of an Fc specific Fab fragment and incubated for 1 h at 37^o^C. Virus containing supernates were added to cells for 3 h at 37^o^C. Cells were rinsed and incubated at 37°C for 96 h. RNA from infected cells was isolated using a RNAeasy Miniprep kit (Qiagen). cDNA was prepared using a Superscript III kit, followed by real-time PCR to quantify enhancement of virus titer. A curve for virus dilutions was used as a comparative control, and 18S RNA was used as an internal control.

For determining the role of specific FcR, cells (1×10^5^) were treated with antibodies directed to respective cellular FcRs (Ancell, Bay Port, MN) at a concentration of 2ug/ml. Cells were incubated for 15 minutes in the presence of 1 ug of each individual FcR-inhibiting antibody prior to adsorption of the virus-antibody mixture. Viral RNA was quantified by real-time PCR (as above) and compared to those of control ADE infections in the absence of an antibody directed specifically against FcR.

### Flowcytometry

Cell surface expression of Fc receptors was quantified by flow cytometry. IHH or Huh7 cells were grown on plastic surface and detached by treatment with Accutase (Sigma), washed with phosphate-buffered saline (PBS), and incubated with antibodies to Fc receptors (CD16, CD32, and CD64 - procured from Ancell, Bayport, MN) at a concentration of 4 ug/ml, followed by the addition of a FITC conjugated secondary antibody for staining. Cells were rinsed and resuspended in methanol free 1% paraformaldehyde in PBS and gated according to their size (forward light scatter) and granularity (side light scatter) using a Becton Dickinson flow cytometer. Surface marker expression on gated cells was analyzed using FlowJo (Tree Star) and CellQuest (BD Immunocytometry Systems) software. The Statistica program was used for analyses of variations.

### Pseudotype virus

VSV derived pseudotypes were generated from BHK cells expressing HCV envelope glycoproteins (E1 and/or E2), with control virus being derived from cells expressing VSV-G protein as previously described [3]. Treatment of VSV/HCV pseudotype with an antiserum to VSV G did not alter virus titer, suggesting the absence of revertant VSV G in pseudotype preparation(s), while positive-control virus incorporating native VSV G exhibited pseudotype neutralization as previously reported. NMSO3 (1.25 µg/ml) was used for an additional safeguard from contaminating VSV G glycoprotein reconstitution on pseudotype virus [3]. The assay for pseudotype infectivity in the presence or absence of antibody was performed in Huh7 cells as described earlier [20]. Parental VSV was exposed to the same experimental conditions for use as a control against nonspecific ADE.

## Results

### Enhancement of HCVcc infection by vaccinee sera

**Figure 1:**
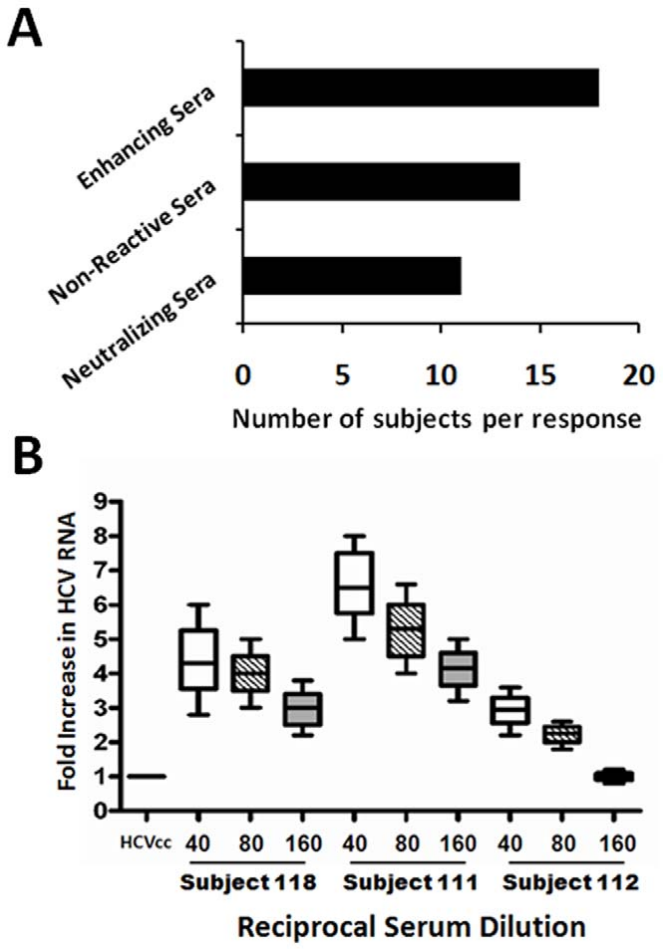
Screening for cell culture grown HCV genotype 1a (clone H77) infectivity in the presence of vaccine sera. The bar diagrams represent the overall outcome of the vaccinee sera to interfere with virus infectivity. Enhancing sera are defined as those which display a >2 fold increase in FFU, while not displaying neutralization at dilutions between 1∶10 and 1∶160. Neutralizing sera are those which display ≥50% decrease in FFU at a dilution of ≥1∶20 (panel A). Enhancement of cell culture grown HCV genotype 1a infectivity using serial dilutions of selected vaccinee sera are shown (panel B). Fold increase in real-time PCR values of HCV RNA with three representative sera at different dilutions, and a decrease in HCV RNA in response to a different vaccinee serum are shown.

Vaccination stimulated low levels of antibodies in subjects [21]. Results obtained from experiments examining the neutralization of cell culture grown HCV (HCVcc) by immunofluorescence (FFU) displayed the number of neutralizing, non-reactive, or enhancing vaccine sera as summarized (Fig. 1, panel A). To further define the ability of non-neutralizing sera to enhance HCV infectivity, we infected IHH with HCV genotype 1a (clone H77) in the presence or absence of heat inactivated HCV specific vaccinee sera. Subsequently, we used a sensitive real-time PCR assay to quantitate HCV genotype 1a genomic RNA copies to demonstrate increased levels of virus after antibody mediated enhancement of HCV infection. The results from three representative sera displaying at least 3 fold increase in HCV RNA at different serum dilutions is shown (Fig. 1, panel B). A separate vaccine sera observed to neutralize both HCVcc and pseudotype was used at the same dilutions and was observed to strongly neutralize virus infectivity at all dilutions tested. Sera from pre-vaccinated subjects did not display any significant reactivity to cell culture grown HCV in these experiments.

**Figure 2:**
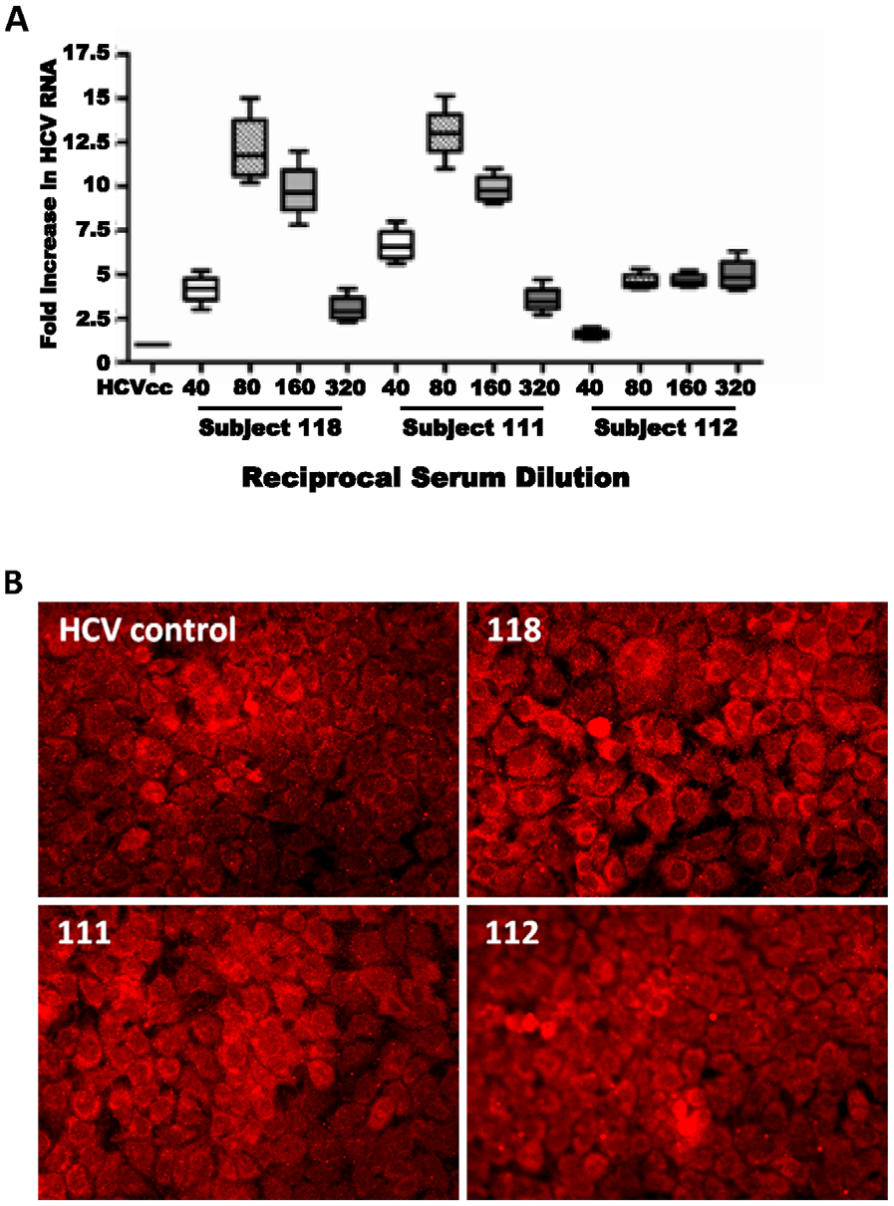
Enhancement of HCVgenotype 2a (clone JFH1) infectivity by serial dilutions of the selected vaccinee sera. A predetermined HCV titer was incubated with vaccinee sera for 1 h prior to addition to an Huh-7.5 cell monolayer for infectivity. Fold increase of HCV RNA by real-time PCR is shown (panels A). Sera were also analyzed for ADE by immunofluorescence, and photomicrographs were captured at 200X magnification (panel B). A predetermined number of FFU were used to infect Huh-7.5 cells in the presence or absence of vaccinee sera at a 1∶80 dilution, followed by 72 h incubation. HCV protein expression was detected using an antibody specific to the HCV core protein, followed by anti-mouse immunoglobulin (Ig) conjugated with Alexa 568 (Molecular Probes, Eugene, OR). Cells infected with virus prior treated with vaccinee sera displayed a greater degree of infection as compared to infected controls in the absence of sera.

The use of cell culture grown HCV JFH1 has become widely accepted virus of choice in understanding diverse aspects of HCV. In our hands, we have observed that the infectious titer of this virus is much higher and the readout is therefore more convenient. Higher growth of the HCV2a clone is likely due to a stronger polymerase activity of the virus [35]. We also examined how these vaccinee sera react with cell culture grown HCV genotype 2a (Fig. 2, panel A). Interestingly, vaccinee sera exhibited a higher level of viral RNA detected upon dilution to 1∶80, which could be attributed to genotype differences. Together, these results display an increased HCV titer or quantity of genomic RNA in hepatocytes in the presence of some of the vaccinee sera, suggesting ADE of HCV infection. A separate vaccine sera observed to neutralize both HCVcc and pseudotype virus was used at the same dilutions and was observed to neutralize virus to higher dilutions (1∶320).

Immunofluorescence to detect the level of FFU(Focus Forming Units) in infected cells was performed to further verify serum generating ADE of HCV JFH-1 infection. Huh7.5 cells infected with virus treated with vaccinee sera prior to cell overlay displayed a distinctly higher level of infection, as evidenced from FFU in all fields analyzed. A representative series of images are shown (Fig. 2, panel B).

### Enhancement of VSV/HCV pseudotype infection by vaccinee sera

**Figure 3:**
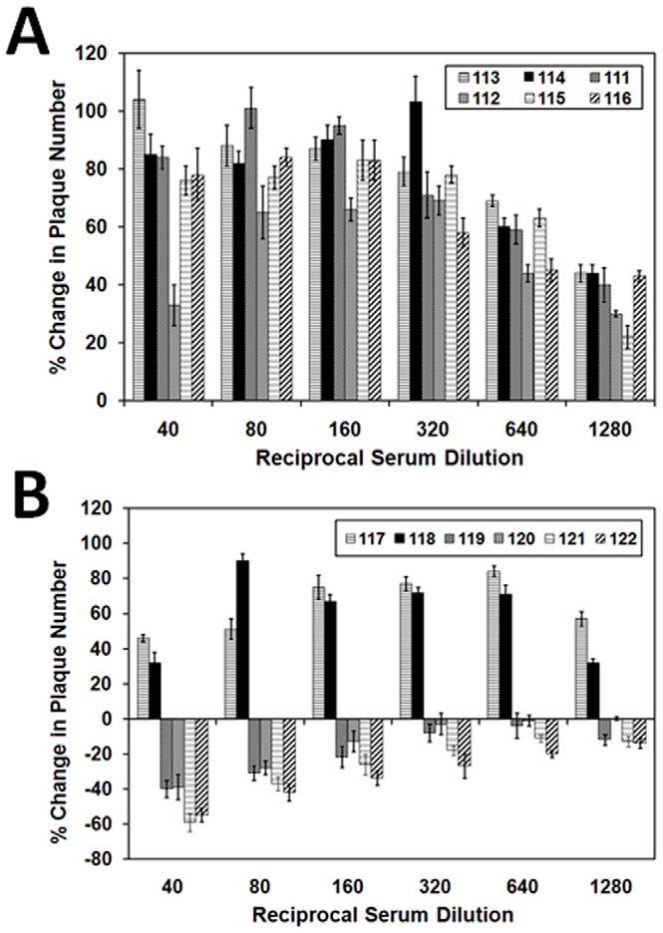
Weak neutralizing activity of vaccinee sera correlates with ADE. Vaccinee sera were selected by their ability to enhance or neutralize of VSV/HCV pseudotype. Sera were serially diluted and incubated for 1h at 37°C prior to infection of Huh-7.5 cells. Sera with neutralizing titers (>1/20) did not exhibit ADE at the dilutions tested.

In earlier studies, we have observed a rise in pseudotype plaque number when serum dilutions fell well below a neutralizing concentration [20]. To further explore this observation, heat inactivated vaccinee sera were tested for their ability to enhance infection at dilutions which fell below their normal neutralizing thresholds in a cell line which is permissive for HCV infection. A pseudotype-vaccinee serum mixture was subjected to plaque titer determination, and the calculated values (percentage of control) are shown (Fig. 3). Sera from healthy uninfected donors enhanced pseudotype infectivity by less than 20%, and this property decreased with increasing serum dilutions (data not shown). Sera from six HCV-vaccinated patients with a poor neutralizing activity exhibited enhancement over a range of serum dilutions (Fig. 3, panel A). Interestingly, representative sera which exhibited the strongest ability to neutralize pseudotype virus (sera # 119, 120, 121, and 122) failed to exhibit any significant enhancing titer (Fig. 3, panel B). However, the ability to successfully neutralize pseudotype virus did not correlate with an inability to also enhance infection at higher dilutions, as two sera which displayed a modest level of neutralization (sera # 117 and 118) were observed to also enhance infection in a similar manner as above. The data from this set of experiments suggested that an inhibition of virus infection occurs when the ratio of neutralizing antibody to virus is high; whereas in a manner similar to that observed in context to dengue hemorrhagic fever, enhancement occurs when this ratio is reversed [36].

### Enhancement of VSV/HCV pseudotype infection by E2 glycoprotein specific antibodies

**Figure 4:**
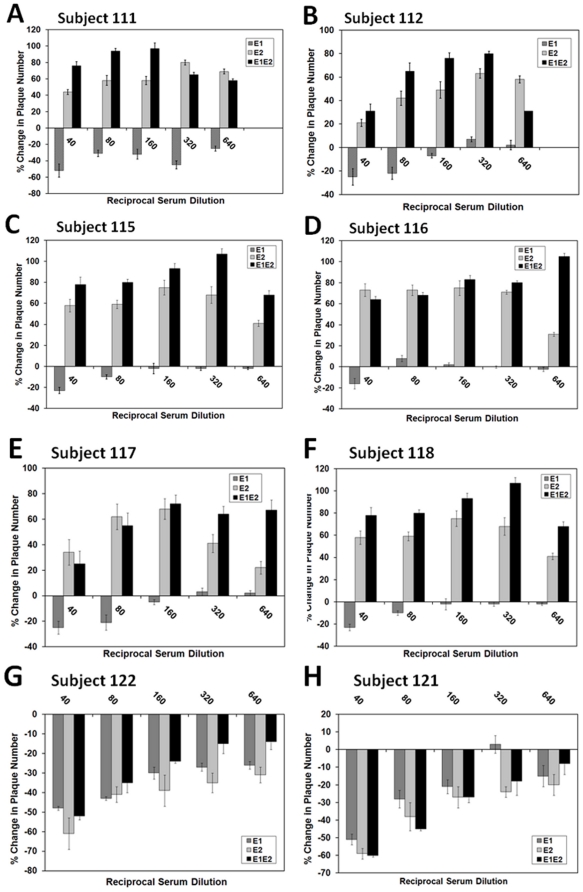
Serum induced enhancement is specific for VSV derived pseudotype bearing HCV E2 glycoprotein. VSV/HCV pseudotype bearing E1 and/or E2 envelope glycoproteins were exposed to serial dilutions of vaccinee sera for 1 h at 37°C prior to infection of Huh-7.5 cells. Antibody dependent enhancement (panels A–F) and neutralization (panels G–H) of different pseudotype viruses are shown.

We have observed a rise in pseudotype plaque number when serum dilutions fell well below a neutralizing concentration. To further examine this observation, vaccinee sera were tested for their reactivity at dilutions which fell below their normal neutralizing thresholds for E1 and/or E2 pseudotype. The use of each individual glycoprotein alone on a VSV backbone exhibited ligand binding unique to the individual glycoprotein; although virus titer was significantly less than that seen when the HCV envelope glycoproteins were both incorporated into the pseudotype virus. However, use of the envelope protein alone on HIV based pseudotype displayed a low level of fluorescence above background (potentially due to poor incorporation of these internally expressed glycoproteins), making the use of this pseudotype difficult. The pseudotype-vaccinee serum mixture was subjected to plaque titer determination, and the calculated values (percentage of control) are shown from sera from 8 subjects (Fig. 4, panels A–H). Six representative vaccinee sera (sera # 111, 112, 115, 116, 117, 118) exhibited an enhancing activity of the pseudotype generated from E2 alone or from E1/E2 covering a broad range of serum dilutions. However, pseudotype generated with E1 glycoprotein alone exhibited some degree of neutralization by all 6 vaccinee sera which displayed ADE with no significant enhancement. As noted above, representative neutralizing sera (sera # 121 and 122) failed to generate an enhancing effect upon any of the pseudotype virus used. The results from this set of experiments suggested that both neutralizing and enhancing epitopes exist on the E2 envelope glycoprotein of HCV, and generation of neutralizing antibodies to E2 may be important for virus neutralization.

### Enhancement of infection is mediated by hepatocyte surface FcR

**Figure 5:**
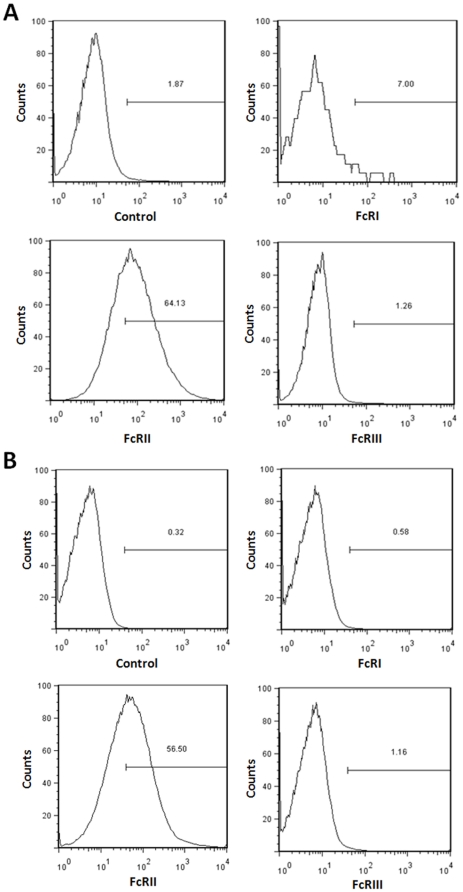
Fc receptor expression on hepatocyte surface. Hepatocytes were reacted with specific antibodies and the expression status of the three primary Fc receptors was analyzed by FACS. Results are shown for IHH (panel A) and Huh-7 (panel B).

**Figure 6:**
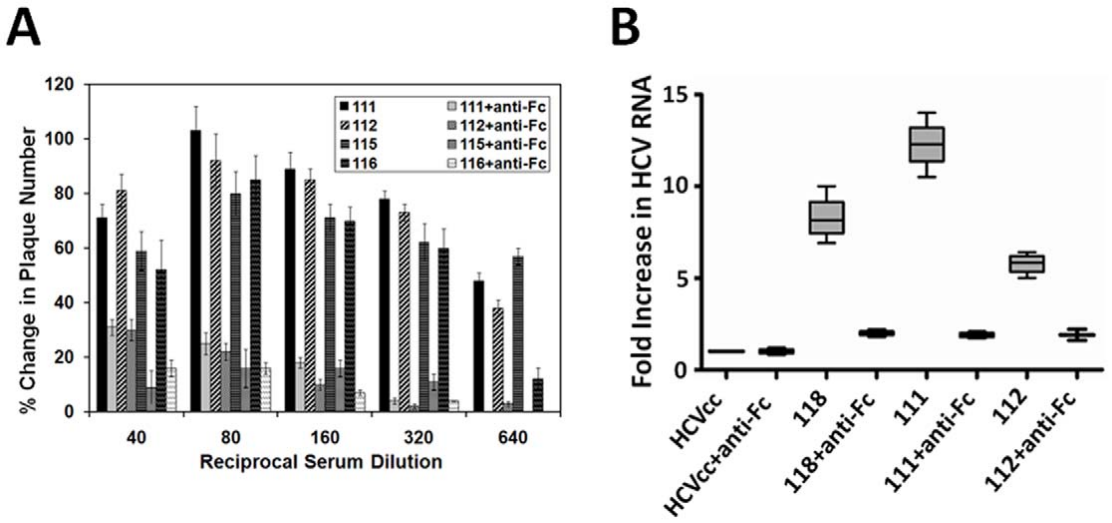
Enhancement of VSV/HCV pseudotype infection via serum addition is dependent upon Fc region of the IgG. A Fab fragment was used to block the attachment of the Fc region of virus adsorbed antibody to hepatocytes subsequent to the binding of the antibody to the pseudotype virus. Non-neutralizing vaccine sera added to pseudotype virus (panel A) or HCVcc (panel B) were treated with 2 mg anti-Fc Fab for 15 minutes prior to addition on Huh-7 cells for infectivity. Virus supernatants treated only with the Fc-Fab fragment were used as controls.

IHH and Huh7 cells were treated with specific antibodies to the Fc receptors (CD16, CD32, and CD64). The expression of FcRII (CD32) was prominent in both cell lines by FACS analysis (56–64%), while expression of the other Fc receptors (CD16 and CD64) was significantly less (<10%) (Fig. 5, panels A–B). FcR-dependent ADE has been demonstrated previously for several viruses. Further, the use of anti-Fc MAbs has been observed to inhibit the enhancing properties of the antibodies tested [37]. Here, the use of an Fc-specific Fab fragment inhibited antibody-mediated enhancement of virus infection by vaccinee sera (Fig. 6, panel A). Further, enhancement of pseudotype infection in Huh7 cells was also inhibited by treatment of the pseudotype/vaccinee serum mixture with 5 µg of anti-Fc (Fab) per ml (Fig. 6, panel B).

**Figure 7:**
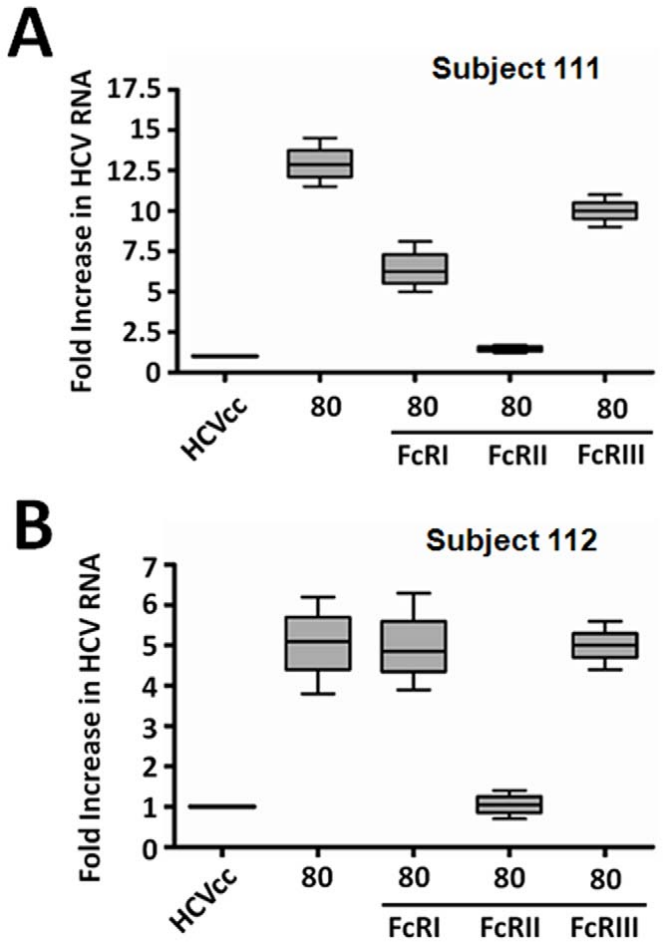
Blocking FcRII on hepatocyte surface leads to inhibition of ADE. HCV was incubated with vaccinee sera for 1 h prior to infection. Huh-7 cells were treated with antibodies known to block the binding activity of the individual Fc receptors 15 minutes prior to infection with the HCV/vaccinee sera mixture. Cells were incubated for 6 h, washed, and incubated for 4 days before RNA preparation and real-time PCR analysis for quantitation of HCV RNA by real-time PCR.

To further define FcR specificity for ADE, cell culture grown HCV was separately incubated with vaccinee sera for 1 h at 32°C. Fifteen minutes prior to infection, cell monolayers were treated with anti-FcR antibodies, and left as competitors after the addition of the virus-antibody mixture. Cells were incubated in the presence of virus for 1 h at 32°C prior to the removal of virus antibody mixture and addition of fresh medium. In cells pretreated with a monoclonal antibody directed specifically to FcRII, percent enhancement attributable to the addition of vaccinee sera was reduced to levels which were similar to those seen in the absence of vaccinee sera (Fig. 7, panels A–B).

## Discussion

We have evaluated the modulation of HCVcc infectivity by serum antibodies taken from vaccinated volunteers. A similar evaluation was made with a VSV/HCV pseudotype system to clarify results derived from cell culture grown HCV and to analyze specific points not observable using HCVcc. This led us to understand the potential for antibody-dependent enhancement of infection, as observed in closely related virus group, i.e. the flaviviruses. Our results suggest that inhibition of virus infection occurs when the ratio of neutralizing antibody to virus is high, whereas enhancement occurs when this ratio is reversed, as observed with dengue hemorrhagic fever [36]. We previously reported the enhancement of VSV/HCV pseudotype infectious titer in the presence of a number of chronically HCV infected patient sera, and with human monoclonal antibodies specific for HCV E2 envelope glycoprotein [20]. A similar evaluation made with human monoclonal antibodies using HCVcc further validated our results. These observations suggested that an enhancement of HCV infection occurs by utilizing a mechanism which is Fc receptor dependent. The nature of HCV specific antibodies generated in human subjects was examined in a susceptible Huh-7 cell line. This phenomenon suggests a potential problem for some viral infections or vaccines that induce low levels of neutralizing antibodies. Antibody-mediated enhancement of infection is sensitive to target cell origin and is dependent on the types and expression levels of Fc receptors on target cell surface [22].

Several viruses elicit antibodies that enhance infectivity through binding of the virus-antibody complex to Fc receptors on cells via the Fc portion of immunoglobulins [24], [38]. This mechanism likely enhances the interaction between the viral envelope protein and its receptor to levels outside those observed during normal infection. Antibody concentrations which fall below levels associated with neutralization have been observed to induce ADE. Sera from individuals infected with HIV-1 displayed an ability to neutralize in vitro infections of HIV-1, but at sub-neutralizing concentrations these same sera enhance infection of permissive cells [39]. Furthermore, the cross protective antibody response of DENV-1 sera for DENV-2 infection was lost at higher dilutions, and displayed ADE [40]. Enhancement of DENV-2 infection has also been noted in a rhesus monkey model using passive transfer of dengue immune sera [31], and an association between increased viral burden and disease severity has been noted [41]. Recently, the use of the rhesus monkey as a model has exhibited similar enhancement using subneutralizing concentrations of a cross reactive monoclonal antibody [42]. ADE in this model could be reduced by altering the Fc region of the antibody to limit binding to Fc receptors, and completely abrogated by a deletion in the CH_2_ region; reinforcing an *in vivo* correlate for ADE. Similarly, passive transfer of DENV-1 specific antibodies into a mouse model subsequently infected with a non-lethal dose of DENV-2 increased the systemic viral burden to multiple tissues and greatly enhanced the mortality rate of infected mice. Antibodies modified to not bind FcR were not able to enhance infection in this model, confirming the importance of specific antibody [43]. Finally, cross reactive prM-specific antibodies for Dengue serotypes have been observed to promote ADE in humans, with enhancement being associated with an increase in the infectivity of poorly infectious virions lacking prM maturation [44]. Immature virus containing unprocessed or poorly processed prM - the precursor viral glycoprotein to the M envelope glycoprotein found in mature, infectious Dengue virions – thereby becomes a secondary source of viral infection.

Interestingly, enhancement of HCV infection by vaccinee sera to genotype 1a glycoproteins appeared to be more prominent for genotype 2a (clone JFH1). Such results may indicate that enhancing epitopes occur across genotypes, while neutralizing epitopes found primarily on the E2 glycoprotein may be more prone to be subjected to selective pressure. The use of a VSV/HCV pseudotype expressing a single glycoprotein from the virus has been used extensively by us [3], [20]. These pseudotyped viruses display extensive differences in ligand sensitivity, and both have incorporated fusion domains in their native sequences. Our results suggest that domains within the E2 glycoprotein are the most likely associated with the enhancement seen in the systems used [20]. Depletion of certain specific competing and/or interfering antibodies may allow systematic evaluation of the roles played by an array of neutralizing antibodies in immune prophylaxis of HCV infection.
